# Osteopontin in the Cerebrospinal Fluid of Patients with Severe Aneurysmal Subarachnoid Hemorrhage

**DOI:** 10.3390/cells8070695

**Published:** 2019-07-10

**Authors:** Maria Giulia Abate, Lorenza Moretto, Ilaria Licari, Teresa Esposito, Lorenzo Capuano, Carlo Olivieri, Arnaldo Benech, Matteo Brucoli, Gian Carlo Avanzi, Gianmaria Cammarota, Umberto Dianzani, Nausicaa Clemente, Gabriele Panzarasa, Giuseppe Citerio, Fabio Carfagna, Giuseppe Cappellano, Francesco Della Corte, Rosanna Vaschetto

**Affiliations:** 1 School of Medicine and Surgery, University of Milan-Bicocca, Milan, Italy, Neurointensive Care, San Gerardo Hospital, ASST-Monza, 20900 Monza, Italy; 2 Department of Translational Medicine, Università degli Studi del Piemonte Orientale, Via Solaroli 17, 28100 Novara (NO), Italy; 3 Anesthesia and Intensive Care, Sant’Andrea Hospital, Mario Abbiate, 21, 13100 Vercelli (VC), Italy; 4 Maxillo facial surgery, “Maggiore della Carità” University Hospital, Corso Mazzini 18, 28100 Novara (NO), Italy; 5 Emergency Department, “Maggiore della Carità” University Hospital, Corso Mazzini 18, 28100 Novara (NO), Italy; 6 Anesthesia and Intensive Care, “Maggiore della Carità” University Hospital, Corso Mazzini 18, 28100 Novara (NO), Italy; 7 CAAD, IRCAD, Department of Health Sciences, Università degli Studi del Piemonte Orientale, Corso Trieste 15, 28100 Novara (NO), Italy; 8 Biochemical Chemistry, “Maggiore della Carità” University Hospital, Corso Mazzini 18, 28100 Novara (NO), Italy; 9 Neurosurgery “Maggiore della Carità” University Hospital, Corso Mazzini 18, 28100 Novara (NO), Italy

**Keywords:** osteopontin, subarachnoid hemorrhage, cerebrospinal fluid, inflammation, biomarker

## Abstract

Aneurysmal subarachnoid hemorrhage (SAH) is associated with high morbidity and mortality. In SAH patients, plasma osteopontin (OPN) has been shown to independently predict poor outcome. The aim of the study is to investigate, in a selected population with severe SAH, OPN time course in cerebrospinal fluid (CSF) and plasma during the first week after aneurism rupture, and OPN prognostic value. We included 44 patients with the following criteria: (1) age 18 and 80 years, (2) diagnosis of SAH from cerebral aneurysm rupture, (3) insertion of external ventricular drain. Plasma and CSF were sampled at day 1, 4, and 8. OPN levels, in CSF and plasma, displayed a weak correlation on day 1 and were higher, in CSF, in all time points. Only in poor prognosis patients, OPN levels in CSF significantly increased at day 4 and day 8. Plasma OPN at day 1 and 4 was predictor of poor outcome. In conclusion, plasma and CSF OPN displays a weak correlation, on day 1. The higher levels of OPN found in the CSF compared to plasma, suggest OPN production within the CNS after SAH. Furthermore, plasma OPN, at day 1 and 4, seems to be an independent predictor of poor outcome.

## 1. Introduction

Aneurysmal subarachnoid hemorrhage (SAH), is one of the most life-threatening diseases, with high mortality and disability rates [1]. Two main mechanisms contribute to poor patient outcome i.e., early brain injury (EBI) and vasospasm with delayed cerebral ischemia (DCI). EBI occurs within minutes after the rupture of an intracranial aneurysm and it seems to be related to the increase of intracranial pressure, decreased cerebral blood flow, and global cerebral ischemia [2]. SAH induced vasospasm is the delayed narrowing of intracranial arteries that takes place between the 4th and the 10th day after aneurysm rupture [3].

EBI and subsequent vasospasm initiate secondary injuries, such as blood–brain barrier disruption, neuroinflammation and oxidative pathways, release of biological active molecules, microembolism, spasm of the arteries and little vessels that ultimately might lead to injury expansion and brain damage [4].

Osteopontin (OPN) is a pleiotropic acidic glycoprotein that was first described as a bone-specific sialoprotein [5] and then as a molecule expressed in activated T cells, therefore called the “early T cell activated gene” (ETA-1) [6].

OPN is expressed in different forms with intracellular and extracellular localization and is involved in several biological processes, such as migration, chemotaxis, adhesion to inflamed tissues, differentiation, phagocytosis, and cellular survival [7,8,9]. OPN is a key molecule in the pathogenesis of acute and chronic inflammation [10], sepsis [11,12,13], tumor progression [9,14] and autoimmunity [15].

At a cerebral level, OPN is a constituent of the extracellular matrix and is synthesized and secreted by microglia under stress condition [16]. Experimental studies showed that OPN is induced in reactive astrocytes and capillary endothelial cells peaking at 72 h after SAH, during the recovery phase of blood–brain barrier [17]. Recent in vivo studies discovered that intraventricular administration [17,18,19] of recombinant OPN as well as post-insult intranasal administration [20] could reduce EBI and cerebral edema, stabilizing the blood–brain barrier and protecting from vasospasm. Only one clinical study so far has investigated the levels of OPN in the blood of aneurysmal SAH patients showing that OPN might predict worse outcomes [21]. Similarly, in patients with ischaemic stroke, OPN has been shown to predict disability [22,23].

Given the above, the aim of the present study is to investigate, in a selected population with severe SAH that needs an external ventricular cerebrospinal fluid (CSF) drainage, how the level of OPN changes in CSF during the first week after aneurism rupture, its relation with OPN plasma level and the possible prognostic value.

## 2. Materials and Methods

### 2.1. Setting and Patients

The study was performed in the intensive care units of the university hospital “Maggiore della Carità”, Novara, Italy between 1 October 2017 and 31 July 2018 and San Gerardo hospital, Monza, Italy between January 2014 and 31 December 2017, according to the principles outlined in the Declaration of Helsinki. The protocol was approved by the Comitato Etico Interaziendale Novara (protocol 891/CE, studio no. CE 115/17) and by the Comitato Etico della Provincia Monza Brianza (Seduta del 22/02/2014, decreto 159 of the 20/03/2014) and consent was obtained for all patients, according to Italian regulation. Inclusion criteria were: (1) age between 18 and 80 years; (2) diagnosis of subarachnoid hemorrhage from cerebral aneurysm rupture; (3) insertion of external ventricular drain. Exclusion criteria were as follows: (1) age less than 18 or more than 80 years old; (2) bleeding occurred more than 24 h before admission; (3) known coagulopathies or antiplatelet or vitamin K antagonist treatment. At hospital entry, the Glasgow coma scale (GCS) i.e., a 15-point scale for comatose patients, was assessed, a computerized tomography (CT) scan was performed and Fisher score, Hunt and Hess score, and World Federation of Neurosurgical Societies (WFNS) grading system were recorded. Angiography or angio-CT were performed to document presence, dimension, and characteristics of patient aneurysm. SAH treatment was carried out according to the best clinical practice. Patients were monitored with transcranial doppler the first day to assess a baseline velocity and from day 4 every day. Any time patients showed clinical worsening or doppler velocity increased, angiography with endovascular nimodipine infusion was considered. Demographic and clinical variables including age, gender, symptoms at onset, comorbidities, and treatment modality (clipping or coiling) were registered.

Transcranial doppler ultrasound (TCD) vasospasm was defined as mild when mean velocity in any vessel reached 120–150 cm/s, moderate if 150–200 cm/s and severe if >200 cm/s.

Outcomes at 3 months were evaluated by the Glasgow outcome scale extended (GOS-E), an 8-point scale, from death to upper good recovery without any disability left, related to SAH and by modified Rankin Scale (mRS) at 3 months post SAH, and was classified as good (mRS 0–2) or poor (mRS 3–6). To obtain this information, trained staff interviewed either patient himself or a close relative.

A control group of 70 healthy subjects were also included and about 10 mL of blood was taken for OPN determination.

### 2.2. Experimental Analysis

Plasma and cerebrospinal fluid (CSF) were sampled at admission, within the first 24 h from the bleeding and at day 4 and 8. Biological samples were centrifuged (2500 rpm for 30 min) and supernatants aliquoted and frozen at a temperature of −80 °C until analyzed. Circulating OPN levels were quantified using a commercially available enzyme-linked immunosorbent assay kit (DuoSet^®^ by R & D Systems; Minneapolis, MN, USA) according to the manufacturer’s protocol. OPN levels were also determined on plasma samples of 70 healthy subjects.

### 2.3. Statistical Analysis

Categorical variables were reported as proportion and percentile while continuous variables were expressed as median ± interquartile range. To compared between good and poor outcomes assessed as good (mRS 0–2) or poor (mRS 3–6) at 3 months, chi-square or Fisher’ s exact test was used for categorical variables, and Mann–Whitney test for continuous variables. When analyzing OPN levels over time, a Friedman test was applied, followed by Dunn’s multiple comparison test. At a same time-point, the difference between OPN in plasma and CSF samples was determined by Wilcoxon matched-pairs signed rank test. After logarithmic transformation due to a not normal distribution of OPN, a regression analysis was used to assess the relation between CSF and plasmatic OPN levels and Spearman’s rank correlation coefficient was used to determine the strength of correlation. We performed a mixed-effect logistic regression to model the relationship between prognosis and OPN in plasma and liquor, measured over time, adjusted for sex. Data analysis was performed using STATA software, version 14.

## 3. Results

### 3.1. Patients

We enrolled 44 patients with the diagnosis, at ICU admission, of severe SAH following the rupture of cerebral aneurism complicated with acute hydrocephalus that needed an external ventricular CSF drainage for acute hydrocephalous or for the gravity of the intracranial bleeding. Twenty-nine patients have been included at the San Gerardo hospital, Monza, Italy, while 15 patients at the Maggiore della Carità hospital in Novara, Italy. Depicted in Table 1, the main characteristics of the overall population and stratified according to the outcome. Median age was 60 (50–69) years old and 64% were of female gender. The aneurism site was equally distributed between anterior, middle and posterior circulation. Overall, 82% of the patients presented a WFNS grades 4 or 5, 70% a Fisher grade 4 and a median GCS of 6 (4–11). When comparing patients with good and poor outcome according to mRS, the only significant difference at ICU admission was found in GCS, being 11 (11–14) in the good and 6 (4–8) in the poor prognosis. ICU and hospital length of stay (LOS) and ICU mortality were lower in SAH patients with good prognosis compare to those with poor prognosis, despite the results not reaching a statistical difference.

### 3.2. OPN Levels in Plasma and CSF

Compared to health controls, SAH patients presented a significant increase of OPN levels in plasma at each time point considered, i.e., 4.5 (2.6–7.3) ng/mL vs. 89 (41–205) ng/mL at day 1 (<0.0001), 129 (61–330) ng/mL at day 4 (*p* < 0.0001) and 175 (65–772) ng/mL at day 8 (*p* < 0.0001).

**Figure 1:**
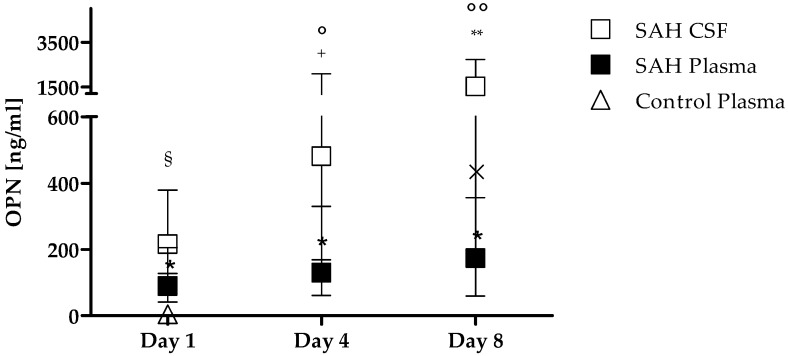
OPN levels in CSF (open square), plasma (solid square) of SAH patients and in plasma of healthy controls (open triangle). Samples from SAH plasma patients at day 1, day 4, and day 8 are significantly higher than plasma levels in healthy controls. Data are expressed as median and interquartile range. * *p* < 0.0001 vs. control plasma, Mann–Whitney test. Samples in CSF and plasma were compared at each time point with Wilcoxon matched-pairs signed rank test. § *p* = 0.0016; + *p* = 0.0005; ** *p* = 0.0002. Plasma OPN levels were compared at day 1 vs. day 4, and at day 8 vs. day 4 with Wilcoxon matched-pairs signed rank test. A similar analysis was performed for CSF samples. In plasma × *p* = 0.046 day 8 vs. day 4; in CSF ° *p* = 0.001 day 4 vs. day 1, °° *p* = 0.034 day 8 vs. day 4. Abbreviations list: OPN: osteopontin; CSF: cerebrospinal fluid; SAH: subarachnoid hemorrhage.

Overall in SAH patients, OPN levels were higher in CSF compared to plasma in all time points i.e., 216 (128–379) vs. 89 (41–205) ng/mL, *p* = 0.0016; 418 (169–2092) vs. 129 (61–330) ng/mL, *p* = 0.0005; 1541 (357–2729) vs. 175 (65–772) ng/mL, *p* = 0.0002, as shown in Figure 1. Moreover, OPN CSF levels significantly increased from day 1 to day 4 (day 4 vs. day 1 *p* = 0.001), and day 8 (day 8 vs. day 4 *p* = 0.034), while in plasma the only barely significant increase was found between day 4 and day 8 (*p* = 0.046) (Figure 1).

After logarithmic transformation, on day 1, we found a significant linear relationship between OPN in plasma and CSF (coefficient regression = 0.331, *p* = 0.042). Plasma OPN was found to correlate with CSF OPN (Spearman’s rho = 0.335, *p* = 0.026). By contrast, no correlation was found on day 4 and day 8.

### 3.3. OPN Levels Stratified by Prognosis

**Figure 2:**
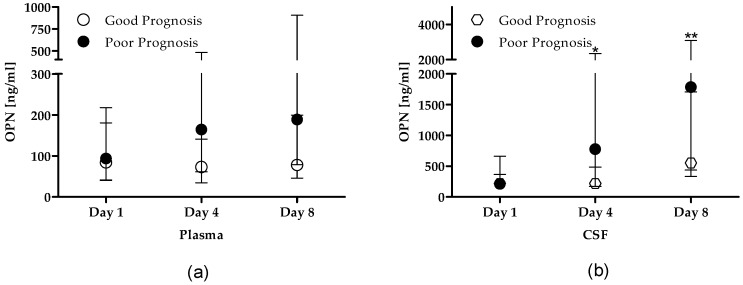
OPN levels stratified by prognosis. OPN levels assessed in 7 patients with good prognosis (open circle) and 37 with bad prognosis (solid circle) in (**a**) plasma samples and (**b**) CSF samples. Data are expressed as median and interquartile range. * *p* < 0.001 vs. day 1, ** *p* < 0.0001 vs. day 1 in CSF samples, Wilcoxon matched-pairs signed rank test. Abbreviations list: OPN: osteopontin; CSF: cerebrospinal fluid.

When stratified patients according to good and poor prognosis, in plasma samples, no difference was found in OPN levels in the time-point considered nor comparing the two prognostic groups (Figure 2a). In CSF samples, OPN dramatically and rapidly raised in patients with poor prognosis, being significant increased already at day 4 compared to day 1 i.e., 735 (170–2296) vs. 213 (124–366) ng/mL, *p* < 0.001) and reaching the highest values at day 8 (1784 (436–3089) ng/mL), when levels were higher than those at day 1 (*p* < 0.0001) but not different compared to day 4 (*p* = 0.130) (Figure 2b). This pattern was not detected in CSF samples of patients with good prognosis. Despite this, no significant difference was found comparing the two prognostic groups.

### 3.4. OPN Levels as Independent Predictors of Poor Prognosis

To determine whether OPN levels at different time points represent an independent risk factor of poor prognosis at 3 months, we constructed a mixed-effect model. In a model comprising gender (male vs. female), OPN plasma level at day 1, 4, and 8, an increase in OPN plasma levels at day 1 (OR: 1.03, *p* < 0.001) and day 4 (OR: 1.12, *p* < 0.001) resulted a risk factor for poor prognosis, while in a model including gender, CSF OPN levels at day 1, 4, and 8, none was associated with poor prognosis (Table 2). Multicollinearity was measured by variance inflation factors and values were in all cases less than 2.

## 4. Discussion

In this observational multicenter study conducted on 44 patients with severe SAH who needed an external ventricular CSF drainage, OPN in CFS displayed a weak though significant correlation with OPN levels in plasma on day 1. Furthermore, OPN levels were persistently higher in CSF compared to plasma, suggesting an active local release and production. Only in patients with poor prognosis, OPN levels in CSF significantly increased at day 4 and day 8 compared to day 1. Finally, our data confirmed, in a selected population with severe SAH, that plasmatic levels of OPN at day 1 and day 4, were independent predictors of poor outcome.

Only one study, to our knowledge, has investigated the role of OPN as a prognostic biomarker in the plasma of SAH patients so far [21]. Nakatsuka et al. enrolled 109 SAH patients and found that plasma OPN independently predicted 90-day poor outcome, suggesting that OPN could be a useful prognostic biomarker in this complex neuro-inflammatory disease [21]. Patients included in their study were less compromised compared to those included in our study, since preoperative WFSN grade 4–5 was present in 29.4% vs. 82%; acute hydrocephalus in 33.9% vs. 100%; mRS 3–6 at 3 months, meaning a poor outcome, of 35% vs. 84% of the patients, respectively. Despite including more severe forms of SAH, we were able to confirm their findings, showing that plasma OPN can be a predictor of poor outcome in our selected population.

In our study, in the overall patients, OPN peaked both in plasma and CSF at 8 days post SAH, but the gradual increase was significant only in CSF. Nakatsuka et al. found a similar kinetic for plasma OPN levels in the overall patients, but when dividing patients according to the complications developed such as acute hydrocephalus or delayed cerebral ischemia, patterns, and peaks were different suggesting that OPN might have a multifunctional role according to the pathophysiology of the diverse phases. Unfortunately, due to the limited number of patients included, we were not able to confirm these data.

Our study is the first to evaluate OPN levels in CSF of SAH patients during the first week after aneurism rupture. We were able to detect a weak but significant correlation between plasma and CSF OPN, but only at day 1. By contrast, a further OPN increase in the subsequent time points was detected in the CSF but not in the plasma with the exception of a barely significant increase from day 4 to 8. This finding suggests that the OPN increase may be due to two distinct biological events. The former might involve the vascular lesion at the interface between the brain and the blood and cause the OPN increase in both compartments. The second might be ascribed to a reactive intrathecal production of OPN and cause OPN increase in the CSF only. In light of this, it is intriguing that only the early OPN increase in the plasma was an independent risk factor for poor prognosis, whereas the late OPN increase in CSF was not, even though it was strikingly higher in the poor prognosis group than in the good prognosis one. This finding suggests that the late OPN increase in the CSF marks severe damage but may also exert some positive effect in tissue protection. In line with this possibility, OPN has been extensively studied in experimental models of SAH in vivo as a potential drug. Pre-treatment with recombinant intraventricular OPN administration [17,18,19] and post-insult intranasal administration [20] prevented EBI and vasospasm, but also stabilized vascular smooth muscle cell phenotype protecting from vasospasm. OPN might also exert a direct effect on microglia by modulating phagocytic activity [24], migration [25], and the release of proinflammatory cytokines [26,27] as seen in different models of cerebral ischemia and neurodegenerative diseases.

OPN levels in CSF were constantly higher than those in plasma, suggesting that, in SAH patients, CSF inflammation is far more intense than the systemic inflammation and that an endogenous production and/or secretion of OPN takes place within the CSF. Other cytokines involved in the pathogenesis of neuro-inflammation behave similarly like the pro-inflammatory cytokine interleukin-6 (IL-6) and the leukocyte chemotactic activating cytokine, IL-8, during hemorrhagic [28,29] and ischemic stroke [30]. The increase in IL-6 levels in CSF after SAH seems to induce the expression of monocyte chemoattractant protein-1 (MCP-1) [31], that correlates to poor outcome [32]. Other biomarkers that have been studied in SAH patients include oxidative stress molecules such as nitric oxide, inflammatory cytokines such as IL-1, tumor necrosis factor-alpha, brain damage biomarkers such as S100B and glial fibrillary acidic protein, and vascular pathology molecules such as endothelin-1 [33,34]. Despite the large number of potential biomarkers, only a few have been studied as possibly therapeutic targets [35].

This study has some limitations that need to be discussed. First, as we selected a limited number of patients who needed insertion of external ventricular drain for clinical purpose—i.e., acute hydrocephalus—our data need to be confirmed in a larger cohort of patients less homogeneous as severity of disease. Second, we observed a peak on day 8, but we did not measure OPN thereafter, although data on literature describe that high OPN levels are stably increased for longer times [21]. Third, we measured only OPN full length in both plasma and CSF, but we can not exclude that other OPN isoforms might have a major role in SAH. Lastly, the exact role and function of OPN in SAH contest remain unknow and new clinical and experimental study need to be done to deep our knowledge on this multifunctional molecule.

## 5. Conclusions

In highly selected patients with hydrocephalus after SAH, intrathecal OPN levels display a weak though significant correlation with OPN plasmatic levels on day 1. The higher OPN levels observed in the intrathecal compartment suggests endogenous cytokine production within the CSF after SAH. Only in patients with poor prognosis at 3 months, OPN levels in CSF significantly increased at day 4 and day 8 compared to day 1. However, only plasmatic levels of OPN at day 1 and day 4 are independent predictors of poor outcome. A larger sample and better distributed throughout the whole SAH clinical spectrum would most probably provide more information on this complex disease.

## Figures and Tables

**Table 1:** Demographic and clinical characteristics of the 44 included patients.

Variable	Good Prognosis mRS 0–2 n = 7	Poor Prognosis mRS 3–6 n = 37	All n = 44	*p* Value
Age, median (IQ)	52 (46–68)	60 (51–70)	60 (50–69)	0.480
Gender, Female	5 (71%)	23 (62%)	28 (64%)	1.000
Ruptured aneurysm location				1.000
Anterior communicating artery and Anterior cerebral artery	2 (29%)	11 (30%)	13 (30%)	
Internal carotid artery	1 (14%)	7 (19%)	8 (18%)	
Middle cerebral artery	2 (29%)	10 (27%)	12 (27%)	
Posterior circulation	2 (29%)	9 (24%)	11 (25%)	
Coiling, n (%)	7 (100%)	27 (73%)	34 (77%)	0.177
Preoperative WFNS grades 4 –5, n (%)	4 (57%)	32 (86%)	36 (82%)	0.100
Fisher grade 4, n (%)	5 (71%)	26 (70%)	31 (70%)	1.000
GCS	11 (11–14)	6 (4–8)	6 (4–11)	0.005
ICU-LOS	15 (13–21)	20 (9–30)	16 (11–28)	0.656
Hospital-LOS	20 (14–21)	26 (10–32)	23 (11–30)	0.610
Vasospasm at day 4 (yes/no)	1/6	5/26	6/32	0.514
Vasospasm at day 8 (yes/no)	2/5	12/18	14/23	0.477
Vasospasm after day 8 (yes/no)	2/5	8/22	10/27	0.612
ICU mortality	0	15 (40%)	15 (34%)	0.077

Abbreviations list: mRS: modified Rankin Scale at 3 months; IQ: interquartile; WFNS: World Federation of Neurological Surgeons scale; GCS: Glasgow Coma Scale; CSF: cerebrospinal fluid; ICU-LOS: intensive care unit length of stay.

**Table 2:** Odds ratio (OR) of poor prognosis at 3 months. Osteopontin plasma concentration and gender as predictors (upper table); CSF OPN concentration as predictor (lower table).

Parameters	OR	95%CI	*p* Value
Gender (Male vs. Female)	2.07	0–16963.21	0.87
Plasma OPN at day 1	1.03	1.01–1.04	<0.001
Plasma OPN at day 4	1.12	1.11–1.14	<0.001
Plasma OPN at day 8	1.00	0.99–1.02	0.56
Gender (Male vs. Female)	1.35	0–48895.24	0.95
CSF OPN at day 1	0.99	0.99–1.01	0.99
CSF OPN at day 4	1.00	1.00–1.00	0.97
CSF OPN at day 8	1.00	1.00–1.00	0.99

Abbreviations list: OR: odds ratio; CI: confidence interval; CSF: cerebrospinal fluid; OPN: osteopontin.
